# Chlorophyll Fluorescence Imaging for Early Detection of Drought and Heat Stress in Strawberry Plants

**DOI:** 10.3390/plants12061387

**Published:** 2023-03-21

**Authors:** Muhammad Akbar Andi Arief, Hangi Kim, Hary Kurniawan, Andri Prima Nugroho, Taehyun Kim, Byoung-Kwan Cho

**Affiliations:** 1 Department of Smart Agricultural System, Chungnam National University, Daejeon 34134, Republic of Korea; 2 Department of Biosystems Machinery Engineering, Chungnam National University, Daejeon 34134, Republic of Korea; 3 Department of Agricultural Engineering, Faculty of Food Technology and Agroindustry, University of Mataram, Mataram 83115, Indonesia; 4 Department of Agricultural and Biosystems Engineering, Faculty of Agricultural Technology, Universitas Gadjah Mada, Yogyakarta 55281, Indonesia; 5 Department of Agriculture Engineering, National Institute of Agricultural Science, Rural Development Administration, Wanju 55365, Republic of Korea

**Keywords:** abiotic stress, chlorophyll fluorescence imaging, strawberry plant, image analysis

## Abstract

The efficiency of photosynthesis in strawberry plants is measured to maintain the quality and quantity of strawberries produced. The latest method used to measure the photosynthetic status of plants is chlorophyll fluorescence imaging (CFI), which has the advantage of obtaining plant spatiotemporal data non-destructively. This study developed a CFI system to measure the maximum quantum efficiency of photochemistry (Fv/Fm). The main components of this system include a chamber for plants to adapt to dark environments, blue LED light sources to excite the chlorophyll in plants, and a monochrome camera with a lens filter attached to capture the emission spectra. In this study, 120 pots of strawberry plants were cultivated for 15 days and divided into four treatment groups: control, drought stress, heat stress, and a combination of drought and heat stress, resulting in Fv/Fm values of 0.802 ± 0.0036, 0.780 ± 0.0026, 0.768 ± 0.0023, and 0.749 ± 0.0099, respectively. A strong correlation was found between the developed system and a chlorophyll meter (r = 0.75). These results prove that the developed CFI system can accurately capture the spatial and temporal dynamics resulting from the response of strawberry plants to abiotic stresses.

## 1. Introduction

Strawberry (*Fragaria × ananassa*) is a crop with significant socioeconomic value worldwide, owing to its organoleptic properties and high nutritional value, as it contains vitamins, minerals, and bioactive compounds such as antioxidants [1]. Therefore, the consumption of strawberries, either fresh or processed, continues to significantly increase, with the market surging every year [2,3,4,5]. Strawberry is one of the most widely grown crops in South Korea, which is a major strawberry-producing country alongside Poland, Mexico, Egypt, Turkey, Spain, and the United States [6,7]. Based on the Korean Statistical Information Service (KOSIS), domestic strawberries generated approximately KRW 1.3 trillion in 2018 [8]. According to the Food and Agriculture Organization (FAO), the production output and area for strawberries in South Korea in 2019 were approximately 200,000 metric tons and 6462 hectares, respectively. According to the South Korean Ministry of Agriculture, Food, and Rural Affairs (MAFRA), strawberry exports hit a record high of USD 65 million in 2021, making it one of the most profitable crops grown. To continue to meet the market demand, it is essential to maintain the productivity of the strawberry plants. However, strawberry plants are prone to various abiotic stresses, such as drought and heat, eventually affecting the plant’s productivity [9,10,11]. Drought conditions affect plant physiology such as photosynthetic responses, transpiration, solute transport, and cellular water status [12,13]. In addition, it has been reported that high temperatures affect strawberry plants, especially during their reproductive processes [14]. Hence, it is necessary to develop a reliable monitoring system that enables non-destructive measurements and provides precise data on the health status of the plants. In addition, a non-destructive technique is more advantageous as it is more flexible for re-testing without damaging the plants [15].

**Figure 1:**
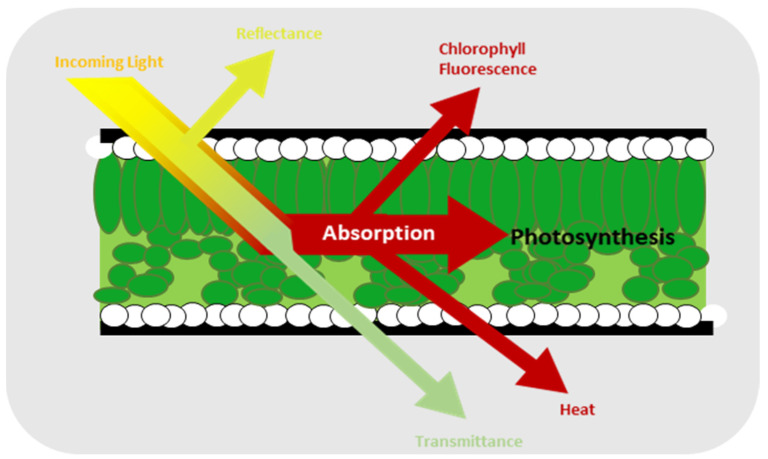
Events caused by light hitting the leaves of a plant [16].

One of the most robust and commonly used methods to evaluate the impact of stressors on plants is chlorophyll fluorescence, which is one of the results of light absorption by plant as shown in Figure 1 [16,17]. Moreover, it is a simple and efficient technique for detecting photosynthesis inhibition [18]. Chlorophyll fluorescence comprises a small fraction of the energy emitted by the chlorophyll molecules from the plant that is not used for photosynthesis [19,20]. Instead, it is used to assess how effectively a plant responds to biotic [21,22,23] and abiotic stress conditions [24,25,26]. It has some parameters such as the ratio of chlorophyll fluorescence decrease (*Rfd*), the Fv/Fm ratio, the efficiency of photosystem II (ϕ*PSII*), the photochemical (*qP*) and non-photochemical (*qN*) quenching of variable chlorophyll fluorescence yield, the relative electron transport rate (*ETR*), and non-photochemical quenching of chlorophyll fluorescence (*NPQ*) [27,28,29]. Of all these parameters, one of the most frequently used for chlorophyll fluorescence is Fv/Fm, which measures the maximum photosystem II (*PSII*) quantum efficiency value and allows for the highly accurate prediction of severe levels due to plant stress [30,31]. In addition, it describes the amount of light absorbed by the chlorophyll in PSII and utilized for photochemical reactions [32]. The formula for Fv/Fm is given by Equation 1, where *Fm* is the maximum fluorescence value after the chlorophyll pigment receives illumination from the light source. Under these conditions, all the electron receptors at the center of the reaction are in a reduced closed state. Fo is the minimum fluorescence value, indicating the ground fluorescence level. In this state, the pigment complex associated with photosystem II reaction centers is assumed to be open. Fv is the difference between Fm and Fo. The value of Fv/Fm for unstressed strawberry leaves has been reported to be greater than 0.79 [33,34].

(1) $$F_{v}/F_{m}=\left(F_{m}-F_{o}\right)/F_{m}.$$

Many commercial systems are used to measure chlorophyll fluorescence, and the most commonly used system is a chlorophyll fluorometer based on pulse amplitude modulation (PAM) or continuous excitation [35]. However, the photosynthetic performance of plants is not homogeneous under stress conditions, and most of these instruments can only measure the chlorophyll fluorescence parameter at the point level on the leaf instead of measuring the spatial variability of the plant canopy [36]. Several measurements must be taken to verify that the evaluation samples represent the entire plant canopy, making the process time-consuming [37]. In addition, many fluorometer instruments require leaf clamps during the measurement process, increasing the risk of damage to the plant sample. To overcome these shortcomings of the fluorometer, chlorophyll fluorescence imaging (CFI), a recent development to quantify chlorophyll fluorescence, is required. Many previous studies have used a commercial CFI system. Although it can measure many parameters of chlorophyll fluorescence at once, using particular electronic components for image capture and processing makes this system more complex, which has implications for its cost. In addition, many commercial CFI systems have low resolution [38]. Therefore, the CFI system developed in this study can be an alternative that is simpler and more affordable as well as having a higher-quality resolution for the early detection of stress in plants. CFI uses pulsed lasers or LED lights to excite the plant and a camera that is usually attached to a lens bandpass filter to capture chlorophyll fluorescence emission from the plant. Chlorophyll fluorescence images are generated by segmenting the captured images and computing the fluorescence parameters for each pixel [39].

To develop an accurate CFI system, instruments are needed to precisely capture the fluorescence signal from strawberry plants. This has implications for determining the light source and bandpass filter, with each functioning, respectively, as a stimulant for excitation and a capturer of plant emission signals. Therefore, it is necessary to investigate changes in the excitation and emission wavelengths of chlorophyll during plant growth by using an emission–excitation (EMEX) matrix. The results of this initial study will be used to develop a CFI system.

This study aims to detect abiotic stresses in strawberry plants using the developed CFI system. Four types of treatments are applied to observe differences in *Fv*/*Fm* values: control, drought stress, heat stress, and a combination of drought and heat. A conventional commercial chlorophyll fluorescence instrument and chlorophyll meter are used to evaluate the developed CFI system to observe how strong the correlation is between them.

Novelty statement: CFI is a non-destructive and highly sensitive technique that can detect early signs of abiotic stress in plants. However, few studies have applied CFI to strawberry plants, which are highly sensitive to abiotic stress.

Hypothesis: It is hypothesized that the developed CFI system can accurately detect abiotic stress in strawberry plants by measuring changes in the *Fv*/*Fm* value.

Aim of the study: This study aims to develop and evaluate a CFI system for detecting abiotic stress in strawberry plants as well as to compare the results with those obtained using a conventional commercial chlorophyll fluorescence instrument and chlorophyll meter. The study also investigates the effects of different types of abiotic stresses (drought, heat, and a combination of both) on the *Fv*/*Fm* value in strawberry plants.

Knowledge gap: While previous studies have investigated the effects of abiotic stress on the photosynthetic efficiency of various plant species using CFI, research on the application of CFI for the early detection of abiotic stress in strawberry plants is limited. This study will help to fill this knowledge gap and provide insights into the use of CFI for monitoring the health and productivity of strawberry plants.

## 2. Materials and Methods

### 2.1. Plant Material and Growth Conditions

In this study, a total of 125 six-month-old strawberry plants were used. The plants were transplanted into cylindrical pots (with a 12 cm diameter and 11 cm height) with the “Hanareum” strawberry nutrient mixture (Shinsung Mineral Co., Ltd., Goesan-gun, Republic of Korea) and placed in a controlled environment room for one week of initial treatment. All strawberry plants were grown for the first week of initial treatment at 22 and 17 °C day- and night-time temperatures, respectively, as well as in 55% RH (relative humidity), and 16/8 h light/dark photoperiods [10]. To prevent drought stress, the pot soil water content was maintained to obtain a 100% volumetric water content (VWC) [11].

### 2.2. EMEX Matrix of Chlorophyll in Strawberry Leaves

After one week of initial treatment, five strawberry pots were placed in the growth chamber under the same treatment conditions for the EMEX matrix measurement. The leaf EMEX matrix was acquired using a spectrofluorometer (SCINCO, FluoroMate FS-2, Seoul, Republic of Korea) to explore changes in the chlorophyll spectrum during the strawberry growth period to build an optimal CFI system. The sample was then placed in a solid sample holder. Five leaf samples from four different pots were collected and measured using a spectrofluorometer for 15 days of observation with intervals of two days each. The excitation wavelength was set as 1 nm increments between 350 and 550 nm, and the emission wavelength was set as 1 nm increments between 600 and 800 nm. The spectral data obtained were processed with the Python 3.9 programming language.

### 2.3. Abiotic Stress Treatment

Four treatment groups, including control, heat stress, drought stress, and a combination of heat and drought stress, were created from the 120 strawberry plants that were placed in two growth chambers after one week of initial treatment. The control group received the same treatment as the initial treatment. The drought stress group was placed in the same growth chamber as the control group but with different watering treatments. Each plant for the drought stress group was maintained at 30% VWC, such that it could be said to be included in the severe drought stress category [40]. The soil moisture content was measured using an EC-5 sensor (Decagon Devices Inc., Pullman, WA, USA). The heat stress and drought–heat combination stress groups were placed in a growth chamber with temperature treatments different from the control and drought stress groups. Both groups suffered heat at 30/25 °C day-/night-time temperatures [10]. In addition, the combined stress group received a drought stress treatment similar to the drought stress group. Measurements were recorded at 2-day intervals for all groups for 15 days.

### 2.4. Relative Chlorophyll Content Measurement

Relative chlorophyll content was measured using a leaf chlorophyll meter (SPAD-502; Minolta Corp., Osaka, Japan). To represent each plant, one daily observed value was obtained by averaging four to five SPAD measurements from all the plants in the four treatment groups; this approach is similar to that used in several previous studies [10,41,42]. Measurements using a chlorophyll meter were conducted every two days after one week of the initial treatment. Measurements were carried out until day 15 of observation.

### 2.5. Chlorophyll Fluorescence Measurement Using a Conventional Commercial System

In this study, the chlorophyll fluorescence of strawberry plants was measured using a chlorophyll fluorometer (Handy-PEA, Hansatech, UK), a commercial instrument used to measure chlorophyll fluorescence parameters, including the *Fv*/*Fm* ratio. Chlorophyll fluorescence measurements were carried out after one week of the initial treatment of the plants, for 15 days of observation with an interval of 2 days between observations. Data collection was carried out by clamping a leaf with a leaf clip, representing a dark adaptation treatment for at least 20 min [34]. Next, the shutter blade on the leaf clip was opened, and the sensor head was attached. Chlorophyll fluorescence was induced by applying a pulse of saturating red light (650 nm peak) [43]. This measurement yielded the values of *Fo*, *Fm*, and *Fv*/*Fm*. The measured chlorophyll fluorescence parameter data were visualized using PEA Plus software.

### 2.6. Chlorophyll Fluorescence Measurement Using the Developed CFI System

**Figure 2:**
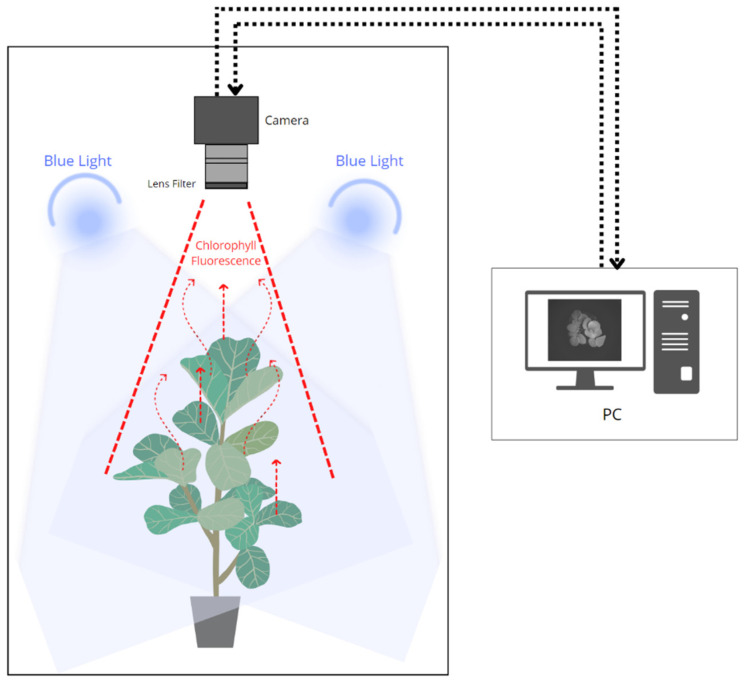
Schematic of the process to obtain fluorescence signal from plant chlorophyll.

In this study, a CFI system was developed to measure photosynthetic efficiency in strawberry plants, represented by one of the chlorophyll fluorescence parameters, namely the maximum quantum efficiency of photochemistry (*Fv*/*Fm*), to detect abiotic stresses in the plants. The CFI method can provide the spatial and temporal dynamics of plant data owing to the stress response. A schematic of this system is shown in Figure 2. In general, this CFI system consisted of a closed chamber with a light source to excite the fluorescence signal (465–470 nm) from chlorophyll after 20 min of dark adaptation. A monochrome camera (MV-CH050-10UM, Hikvision, Hangzhou, China) with a 2448 × 2048 spatial resolution was placed on the top side to capture plant images from the top angle and was equipped with a bandpass filter (BP695, Midwest Optical Systems Inc., Palatine, IL, USA) that provided a range of 680–720 nm to transmit only the fluorescence signal from chlorophyll. This system was designed to measure *Fv*/*Fm*, one of the parameters of chlorophyll fluorescence, based on a patent that uses a similar technique [44].

### 2.7. Image Analysis

**Figure 3:**
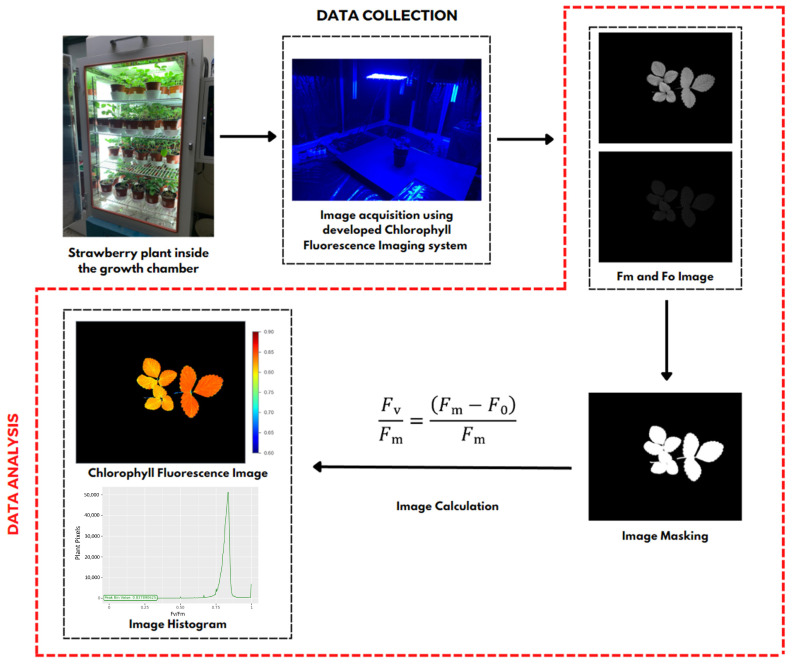
Workflow to develop chlorophyll fluorescence image. The *Fm* and *Fo* images captured using the developed system are processed computationally, which includes masking and calculating the chlorophyll fluorescence parameters to generate chlorophyll fluorescence images along with their histogram images.

After obtaining the fluorescence data, namely *Fm* and *Fo* images, image processing was conducted using a workflow, as shown in Figure 3. All image processing in this study used several Python library functions that aimed to develop chlorophyll fluorescence images and their *Fv*/*Fm* ratio values. Image processing was begun by inputting the image data, which included the maximum chlorophyll (*Fm*) and minimum chlorophyll (*Fo*). Next, a masking process was performed to separate the plant objects processed as foreground and other objects as background. Image masking was performed using the thresholding method, with a value of 1 representing a white pixel with an intensity of 255 as the foreground and a value of 0 representing a black pixel with an intensity of 0 as the background, to produce a binary image. The next step was to calculate *Fv*/*Fm* for each pixel, which was then colored using pseudocolor to produce a chlorophyll fluorescence image of a strawberry plant and a histogram with the peak representing the *Fv*/*Fm* value, which had the highest number of pixels in the plant object.

### 2.8. Statistical Analysis

Standard statistical methods were used in SPSS (SPSS Version:22.0.0, IBM, New York, NY, USA) for data evaluation, including one-way analysis of variance (ANOVA) with a probability level of 0.05. In addition, the Student–Newman–Keuls (SNK) test was used to assess significant differences between the data.

## 3. Results

### 3.1. Emission-Excitation (EMEX) Matrix

**Figure 4:**
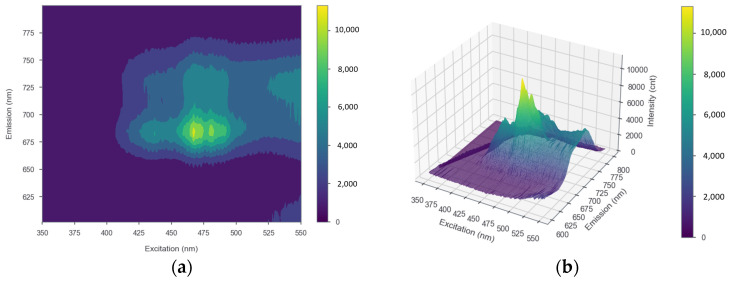
(**a**) 2D and (**b**) 3D EMEX matrices of strawberry leaves as a result of measurements using a spectrofluorometer. The x, y, and z axes show excitation, emission, and intensity, respectively.

The results of measuring strawberry plant leaf samples using a spectrofluorometer, processed into both 2D and 3D EMEX matrices, are shown in Figure 4a,b, respectively. The plot has three axes representing the emission, excitation, and intensity data, with the peaks of the plot indicating the optimum excitation and emission wavelengths. The results of the entire EMEX matrix during the observation period using the SNK test indicated that there was no significant change in the optimal excitation and emission wavelengths. The excitation and emission wavelengths were obtained at approximately 467–469 and 685 nm, respectively.

### 3.2. System Evaluation

**Figure 5:**
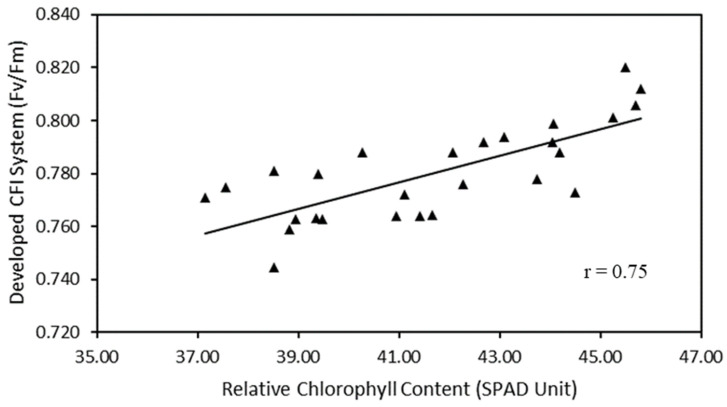
Scatter plots of relative chlorophyll content versus developed CFI system.

Figure 5 shows that the developed CFI system has a strong correlation with the SPAD chlorophyll meter. The correlation coefficient results between the developed CFI system and the SPAD chlorophyll meter have a correlation coefficient of 0.75.

### 3.3. Chlorophyll Fluorescence Imaging

**Figure 6:**
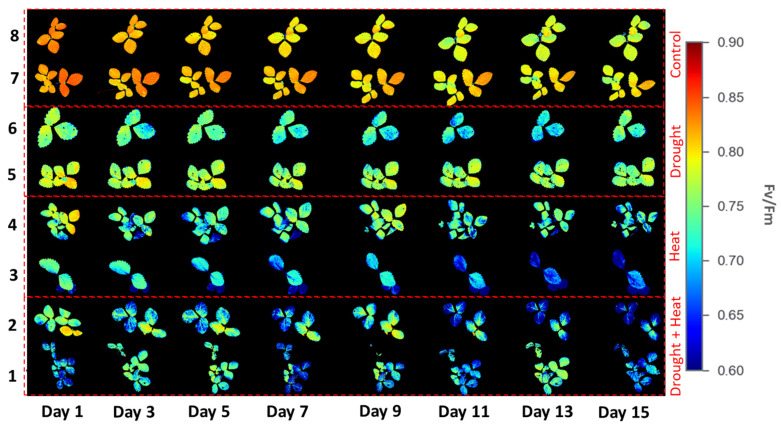
Chlorophyll fluorescence images from four different treatment groups.

Figure 6 illustrates the results of applying four different treatment groups to obtain the *Fv*/*Fm* values of strawberry plants during the 15-day observation period using the developed CFI system. There were color differences between the four groups, indicating different *Fv*/*Fm* values. For example, the control group had a reddish color, indicating a high *Fv*/*Fm* value. By contrast, the plants in the abiotic stress group exhibited a color from green to blue, indicating a low *Fv*/*Fm* value. These differences were observed on the first day of the observation.

### 3.4. Fv/Fm Value from the Developed CFI System

**Figure 7:**
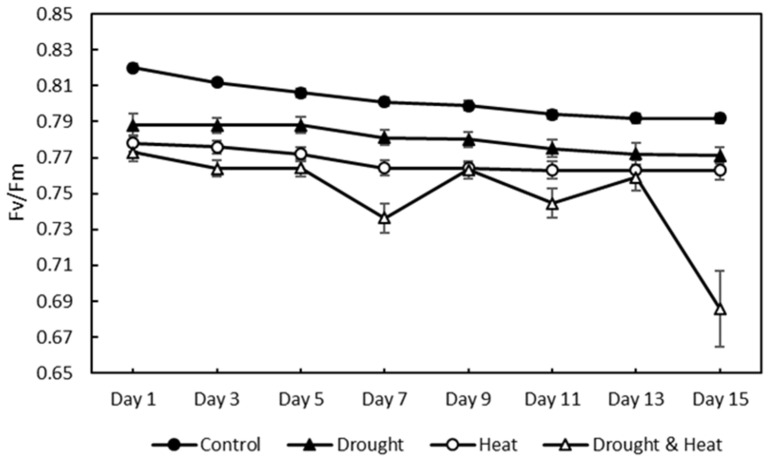
Effect of four treatments (control, drought, heat, and combination drought–heat stress) during 15 days of observation on the maximum quantum efficiency of photochemistry (*Fv*/*Fm*) value using the developed CFI system. The vertical bar represents the standard error (SE) for 30 replications.

**Figure 8:**
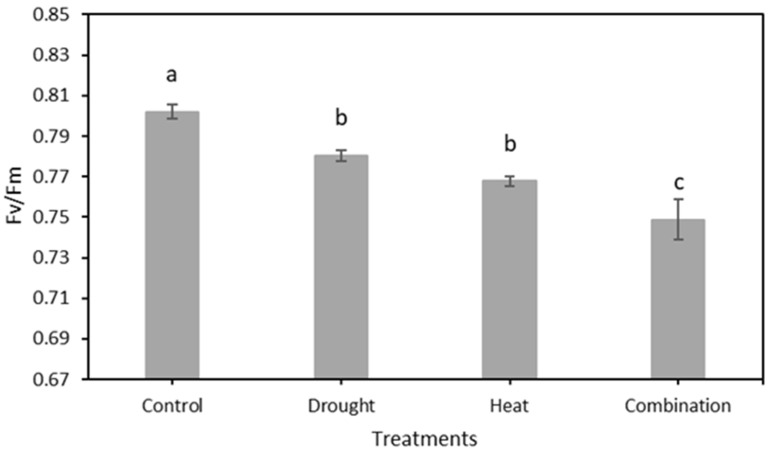
The mean value of *Fv*/*Fm* from the total observation period in four treatment groups using the developed CFI system. The SNK test showed no significant difference between the mean represented by the same letter at *p* ≤ 0.05.

The chlorophyll fluorescence results, as indicated by the *Fv*/*Fm* value of 120 strawberry plants divided into four treatment groups for 15 days of observation, are shown in Figure 7. The graph shows that there was a decrease in the value of *Fv*/*Fm* in all four treatment groups. The SNK test results in Figure 8 show a significant decrease in *Fv*/*Fm* values in the three abiotic stress groups, namely drought (0.780 ± 0.0026), heat (0.768 ± 0.0023), and drought–heat combination (0.749 ± 0.0099), compared with the control group (0.802 ± 0.0036). During the 15 days of observation, the *Fv*/*Fm* value for the control plant group was still in the range of values greater than or equal to 0.79, which is the limit value for healthy plants, whereas the three abiotic stress treatment groups had *Fv*/*Fm* values of less than 0.79 since day 1 after treatment.

### 3.5. Relative Chlorophyll Content

**Figure 9:**
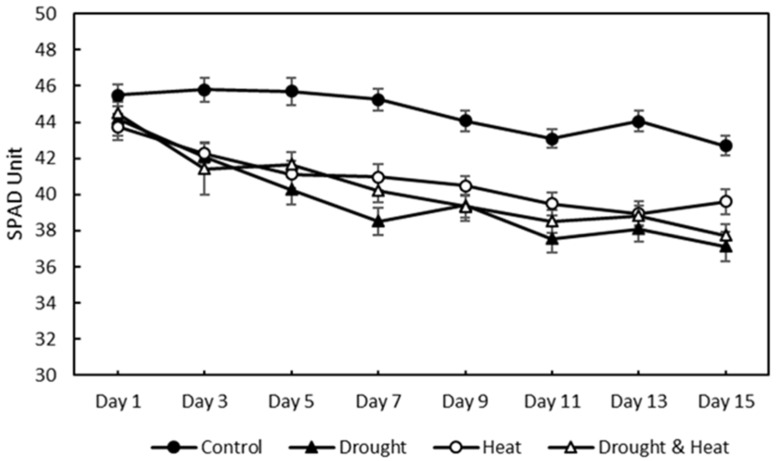
Effect of four treatments (control, drought stress, heat stress, and combination drought–heat stress) during 15 days of observation on relative chlorophyll content (SPAD unit) value using chlorophyll meter. The vertical bar represents the standard error (SE) for 30 replications.

After 15 days of treatment, the relative chlorophyll content was measured using a chlorophyll meter, as shown in Figure 9. The four treatment groups showed a decreasing trend in the relative chlorophyll content. However, the control treatment group had a higher SPAD value than the other three treatment groups, with maximum and minimum values of 45.81 and 42.68, respectively. The maximum–minimum SPAD values for the drought, heat, and combination stress treatment groups were 44.19–37.15, 43.74–38.95, and 44.50–37.73, respectively.

## 4. Discussion

The CFI system developed in this study is based on the characteristics of the chlorophyll spectra of strawberry plants, measured using a spectrofluorometer. Using the SNK test with α = 0.05, it was found that there was no meaningful change in the excitation and emission wavelengths during the observation period. This result indicated that there was no significant change in the chlorophyll spectra during the growth period of the strawberry plants, which correlated with the study in [45] which also determined the chlorophyll spectra of *Triticum aestivum* or common wheat plants using a spectrofluorometer instrument, and the study in [46], which stated that chlorophyll is excited by wavelengths ranging from UV, blue, or green light and emits a fluorescence signal in red with a maximum of 685 nm. These results can be applied to develop a CFI system to determine the excitation light and use appropriate camera filters to obtain chlorophyll fluorescence data for strawberry plants.

Furthermore, information on the physiological status of plants can be obtained by measuring their chlorophyll fluorescence using conventional hand-held instruments. Although conventional instruments can rapidly measure the status of light-dependent reactions, they cannot provide spatial data on the entire plant. This drawback can be overcome by using an imaging system approach. In this study, a CFI system was developed to provide spatial and temporal data on strawberry plants. A strong correlation was observed between the developed imaging system and measurements using the SPAD chlorophyll meter. Moreover, this result was confirmed in several previous studies, which explained that a decrease in SPAD value indicated reduced chlorophyll content owing to damage from PSII caused by abiotic stress [47,48,49]. According to one study [50], a decrease in plant chlorophyll content can impair plant physiological performance, particularly the rate of photosynthesis (Pn) and stomatal conductance (Gs), leading to reduced plant productivity and suboptimal growth. When chlorophyll content is reduced, plants face difficulties in capturing and utilizing light energy for photosynthesis, which reduces the production of carbohydrates necessary for growth and development. Furthermore, a decrease in Gs can limit the access of a plant to the carbon dioxide required for photosynthesis, further decreasing the rate of photosynthesis. In addition, the study from [41] reported that the SPAD value could decrease with an increase in drought stress.

The chlorophyll fluorometer and SPAD chlorophyll meter are similar in that their measurement scale is limited to the point level on the leaf. However, the developed CFI system can cover a wider area of crops. As photosynthetic efficiency, which has implications for chlorophyll fluorescence values, is not homogeneous across plants, differences in spatial data measures between the developed CFI system and the two commercial hand-held instruments affect the correlation coefficient values. Based on similar studies testing CFI systems and hand-held instruments, it can be inferred that obtaining full canopy data using the hand-held system is time-consuming. However, a CFI system can provide data significantly faster [37].

**Figure 10:**
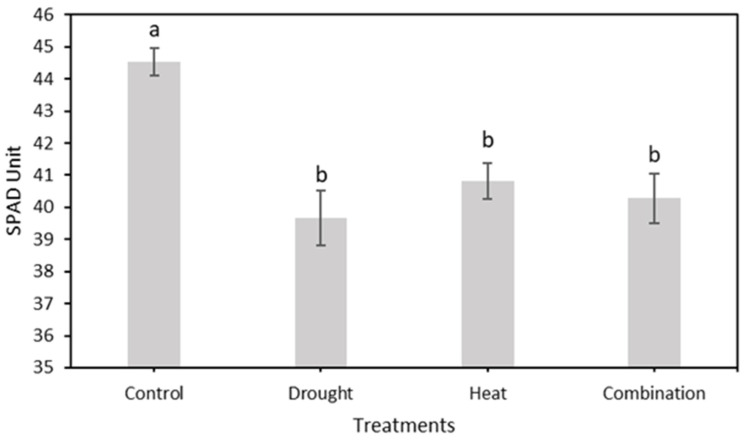
The mean value of relative chlorophyll content (SPAD value) from the total observation period in four treatment groups using a conventional system. The SNK test showed no significant difference between the means represented by the same letter at *p* ≤ 0.05.

In this study, strawberry plants were divided into four groups: control, drought stress, heat stress, and drought–heat combination groups. During the 15 days of chlorophyll fluorescence observation, there was a decrease in *Fv*/*Fm* values in all treatment groups (Figure 7). Studies have reported that the application of drought and heat stress can have significant impacts on plants, affecting their physiology, morphology, and biochemistry, including plant photosynthetic efficiency [51,52]. To determine whether there were significant differences between treatment groups, the SNK statistical test was performed with a significance level of 0.05. The results of *Fv*/*Fm* measurements and SPAD values, presented in Figure 8 and Figure 10, respectively, demonstrate significant differences between the control and stressed plant groups, consistent with the findings of the study in [53], which employed a similar stress treatment to the one used in this study. In the abiotic stress group, the decrease in the value of the *Fv*/*Fm* ratio was caused by the damage to photosystem II (PSII) as a result of the treatment applied, similar to the results obtained in previous studies [33,34,53]. Moreover, a decrease in the *Fv*/*Fm* ratio was observed in the control group, although the difference was not as significant as those in the other groups. This decrease occurs because of aging in plant leaves, as described in a previous study [54]. Previous studies have also found that the *Fv*/*Fm* ratio for strawberry plants under stress conditions was less than 0.79 [33,34]. Therefore, the developed imaging system can detect the presence of abiotic stress in the three treatment groups (characterized by *Fv*/*Fm* values below 0.79) from the first day of observation. This ratio value indicates that the developed CFI system can aid in the early detection of stress in strawberry plants. Moreover, this result is attributed to the development of this system based on the *Fv*/*Fm* parameter, which is a sensitive indicator of the initial response to plant stress [55].

Previous studies, such as [34], have used CFI techniques to measure the photosynthetic activity of a single leaf in a strawberry plant by using differences in light intensity and temperature. Their results showed that high ambient temperatures could cause stress conditions in strawberry plants, leading to lower *Fv*/*Fm* values. This finding is consistent with the results obtained in the present study. Furthermore, several previous studies have only measured the photosynthetic activity of specific parts of a plant, such as a single leaf [33,34,56]. In contrast, this study addresses this gap by providing spatial data on the photosynthetic activity of the entire plant, thus opening up the potential for further research to investigate the distribution of photosynthetic activity in plants.

However, this study is limited in scope to measuring chlorophyll fluorescence only in strawberry plants using *Fv*/*Fm* parameters. Furthermore, the stress applied to strawberry plants is limited to abiotic stress. In addition to the aforementioned limitations, this study was conducted on relatively small-sized strawberry plants that were individually potted and only 6 months old, which may not be representative of the conditions of larger canopy scales. Moreover, the manual measurement process employed between the pots was not efficient, which could have impacted the accuracy and reliability of the results. To address this issue, a conveyor system, as demonstrated in the study in [57], improved the time efficiency of the measurement process as well as enhanced the accuracy and precision of the results.

The developd CFI system has great potential to be applied to various types of plants, but preliminary studies are needed to determine the chlorophyll characteristics of the plants to be tested to obtain accurate data. In addition, the image processing system can be improved to obtain real-time chlorophyll fluorescence images. The CFI system also has the potential to be combined with other imaging techniques, such as the use of LIDAR in the study in [58] and the combined use of CFI and laser-based spectroscopy in the study in [59]. Further research and development can be done to improve the CFI system, such as improving the data processing and analysis techniques. The findings of this study could also have implications for the broader field of plant science, particularly in improving the understanding of plant responses to abiotic stress and the potential for early stress detection under various environmental conditions. The results of photosynthetic activity obtained from this system can help optimize crop yields practically, by allowing the selection of plant variants with resistance to various stresses or by detecting stress as early as possible to assist farmers or stakeholders in their decision-making.

## 5. Conclusions

This study developed a CFI system, a non-destructive technique, to measure the maximum quantum efficiency of photochemistry (*Fv*/*Fm*) as a parameter of photosynthetic efficiency to detect abiotic stresses in strawberry plants. The excitation and emission spectra characteristics reflected in the EMEX matrix plot were measured using a spectrofluorometer to develop a system that can accurately detect chlorophyll fluorescence in strawberry plants. Statistical results using the SNK test indicated that there was no significant change in the excitation and emission wavelengths of strawberry plant chlorophyll during the observation period, with the optimal excitation and emission wavelengths obtained at approximately 467–469 and 685 nm, respectively. The CFI system developed in this study aimed to non-destructively detect abiotic stresses in strawberry plants during a 15-day growth period. A total of 120 pots of strawberry plants were cultivated for one week as the initial treatment and then divided into four treatment groups: control, drought stress, heat stress, and a combination of drought and heat stress. The developed CFI system used a blue LED light (465–470 nm) as a light source to excite chlorophyll and a monochrome camera with a bandpass filter (680–720 nm) to capture the fluorescence emission of strawberry plants after a 20 min dark adaptation process. A strong positive correlation was found between the developed CFI system and the SPAD chlorophyll meter (r = 0.75).

The results of the *Fv*/*Fm* measurements of the strawberry plants in this study prove the performance of the developed CFI system. The proposed system measures accurately and provides data to help detect early decreases in photosynthetic activity due to various abiotic stress treatments. This statement is reflected in the results obtained for the abiotic stress detection in the treatment groups from the first measurement day. It is hoped that the application of this system will help increase the productivity of various crops, especially by preventing plants from being exposed to various types of stress. The developed system can also be a simpler and more affordable alternative to obtain spatial and temporal plant photosynthetic activity data compared to existing commercial systems. Furthermore, the high correlation between the imaging system and commercial instruments supports the accuracy of the acquisition of the photosynthetic status of strawberry plants. Increasing the accuracy and speed of data processing needs to be considered for the further development of this system. The study is limited to measuring photosynthetic activity only in strawberry plants using *Fv*/*Fm*, which is one of the parameters of chlorophyll fluorescence. In addition, the various stresses applied are limited to the category of abiotic stress. Going forward, this system has the potential to be applied in the field, such as in greenhouses, by implementing a mobile system that is integrated with the CFI system.

## Data Availability

All the necessary data were contained in the paper.
